# *α*-Glucosidase Inhibitory Effect and Simultaneous Quantification of Three Major Flavonoid Glycosides in *Microctis folium*

**DOI:** 10.3390/molecules18044221

**Published:** 2013-04-10

**Authors:** Yan-Gan Chen, Ping Li, Peng Li, Ru Yan, Xiao-Qi Zhang, Ying Wang, Xian-Tao Zhang, Wen-Cai Ye, Qing-Wen Zhang

**Affiliations:** 1 State Key Laboratory of Quality Research in Chinese Medicine, Institute of Chinese Medical Sciences, University of Macau, Macao, China; E-Mails: mb05840@umac.mo (Y.-G.C.); mb05817@umac.mo (P.L.); pengli@umac.mo (P.L.); ruyan@umac.mo (R.Y.); 2 Institute of Traditional Chinese Medicine and Natural Products, Jinan University, Guangzhou 510632, China; E-Mails: xqzhang7401@yahoo.com.cn (X.-Q.Z.); chywc@yahoo.com.cn (W.-C.Y.); 3 Guangdong Research Institute of Traditional Chinese Medicine, Guangzhou 510520, China; E-Mail: zxtcpu@yahoo.com.cn

**Keywords:** *Microctis Folium*, flavonoid glycosides, *α*-glucosidase inhibitory effect, HPLC-DAD, quantification

## Abstract

*Microctis Folium*, the leaves of *Microcos paniculata* L., is a commonly used herbal tea material. The methanol extract of *Microctis Folium* and its principle compounds vitexin (**1**), isovitexin (**2**) and isorhamnetin 3-*O*-*β*-d-rutinoside (**3**) were investigated for their *α*-glucosidase inhibitory effects. The extract showed strong *α*-glucosidase inhibitory effect (IC_50_ = 61.30 μg/mL) and the three flavonoid glycosides **1**–**3** exerted satisfactory *α*-glucosidase inhibitory effects, with IC_50_ values of 244.0 μM, 266.2 μM and 275.4 μM, respectively. A simple and reliable HPLC-DAD method was developed for the quantification of the three flavonoid glycosides in *Microctis Folium* and applied successfully to determine contents of these components in samples collected from different locations. This study suggested that *Microctis Folium* may be a promising candidate for development of herbal antidiabetes drugs, and vitexin, isovitexin and isorhamnetin 3-*O*-*β*-d-rutinoside can be the biomarkers and chemical markers for this plant substance.

## 1. Introduction

Diabetes mellitus is a chronic disease which leads to increased concentrations of glucose in the blood (hyperglycaemia) and over time causes serious damage to many of the body's systems, especially the nerves and blood vessels [1]. The number of people with diabetes has increased from 153 million in 1980, to 347 million in 2008 [2]. The WHO projects that diabetes will be the seventh leading cause of death globally in 2030 and the deaths will double between 2005 and 2030. One therapeutic approach for diabetes is to suppress postprandial hyperglycaemia by inhibition of carbohydrate-hydrolyzing enzymes, such as *α*-glucosidase, which plays a significant role in the regulation of blood glucose levels in the human body. *α*-Glucosidase is the key enzyme catalyzing the final step in the digestive process of carbohydrates. Inhibitors of *α*-glucosidase retard the liberation of D-glucose from complex dietary carbohydrates and delay glucose absorption, thus reducing plasma glucose levels and suppressing post-prandial hyperglycaemia [3]. Nowadays, some *α*-glucosidase inhibitors, e.g., acarbose and voglibose, are widely used clinically to control blood glucose levels of patients. Although effective, they usually cause negative effects such as abdominal distension, flatulence, meteorism and possibly diarrhea [4]. Based on that, many efforts have been made to search for more effective and safer *α*-glucosidase inhibitors, and many natural *α*-glucosidase inhibitors have been found in different materials [5,6,7,8]. On account of their unmatched chemical diversity and biological relevance, natural materials have been widely recognized as potential chemical pools in drug screening. Therefore, the discovery of *α*-glucosidase inhibitors from natural materials such as food matrices in the development of physiological functional food or lead compounds for treatment of diabetes is a promising topic [9,10].

*Microcos paniculata* L., belonging to family *Tiliaceae*, abounds in South China, especially in Guangdong Province. *Microctis Folium*, the dried leaves of *M. paniculata*, is a traditional Chinese medicine used for the treatment of colds, abdominal pain, sore throat and jaundice [11,12]. It is one of the major ingredients in some popular herbal teas sold on the China market. Previous studies indicated that the extract of *Microctis Folium* exerts beneficial pharmaceutical effects on fever, pain, coronary heart disease, angina pectoris, jaundice, digestion, and blood lipid levels [13], and the antidiabetes effects in clinic of a Chinese compound formula containing *Microctis Folium* was also reported [14]. Nevertheless, the impact of *Microctis Folium* alone on diabetes remains unknown and the identity of specific compound(s) responsible for the effect need further confirmation.

Related phytochemical investigations on *Microctis Folium* have been carried out, revealing the presence of flavonoids [15], triterpenes [16], and alkaloids [17]. Flavonoids were confirmed as the main constituents in *Microctis Folium* [16] and they showed various significant bioactivities, including *α*-glucosidase inhibition [18]. Among them, vitexin, isovitexin and isorhamnetin 3-*O*-*β*-d-rutinoside were already reported to possess *α*-glucosidase inhibitory activity *in vivo* or *in vitro* [19,20]. Considering their high contents and bioactivities, these three flavonoid glycosides might also be potential biomarkers and chemical markers for the study of *Microctis Folium.*

As a part of an ongoing project of chemical investigation and analysis of edible herbs from the south of China [21,22,23,24], chemical separation and investigation of *α*-glucosidase inhibitory effects were performed on *Microctis Folium*. A high performance liquid chromatography-diode array detector (HPLC-DAD) method and optimal extraction conditions were also developed and validated for the simultaneous quantitative determination of the bioactive flavonoid glycosides vitexin (**1**), isovitexin (**2**) and isorhamnetin 3-*O*-*β*-d-rutinoside (**3**) in *Microctis Folium.*

## 2. Results and Discussion

### 2.1. Separation and Structural Elucidation

**Figure 1:**
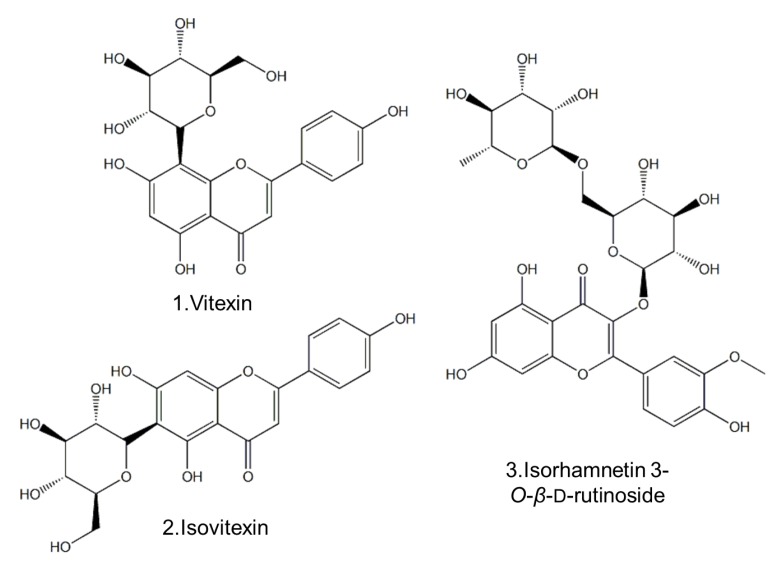
Chemical structures of the three flavonoid glycosides **1**–**3**.

Compounds **1**, **2** and **3** were isolated from the 70% methanol extract of *Microctis Folium* by column chromatography and identified as vitexin (**1**), isovitexin (**2**) and isorhamnetin 3-*O*-*β*-d-rutinoside (**3**), respectively, by comparing their ^1^H-NMR, ^13^C-NMR and MS spectroscopic data with those reported in the literature [16]. The chemical structures of these three flavonoid glycosides are shown Figure 1. 

2.2. α-Glucosidase Inhibitory Effect

**Figure 2:**
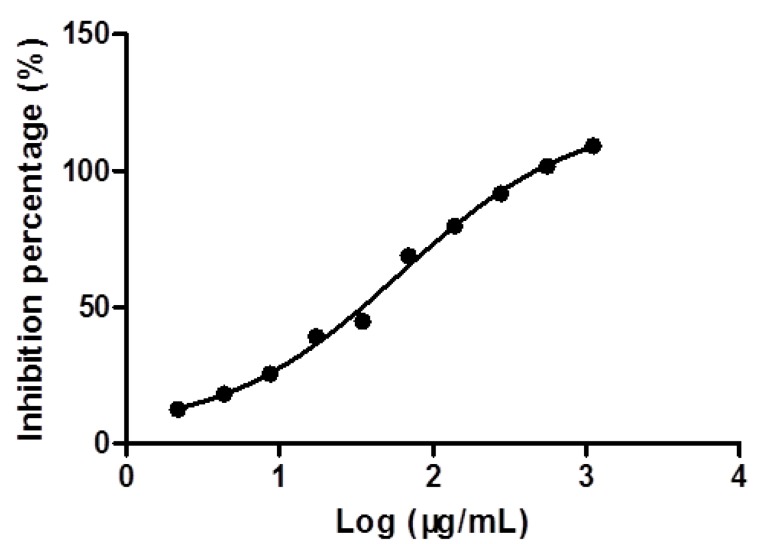
*α*-Glucosidase inhibitory effect of 70% methanol extract of *Microctis Folium*.

The *α*-glucosidase inhibitory activity of the 70% methanol extract of *Microctis Folium* was determined at a series of suitable concentrations (Figure 2). It showed appreciable *α*-glucosidase inhibitory effect in a concentration-dependent manner with an IC_50_ value of 61.30 μg/mL. The result suggested that the extract of *Microctis Folium* might be a promising anti-diabetes drug candidate.

**Figure 3:**
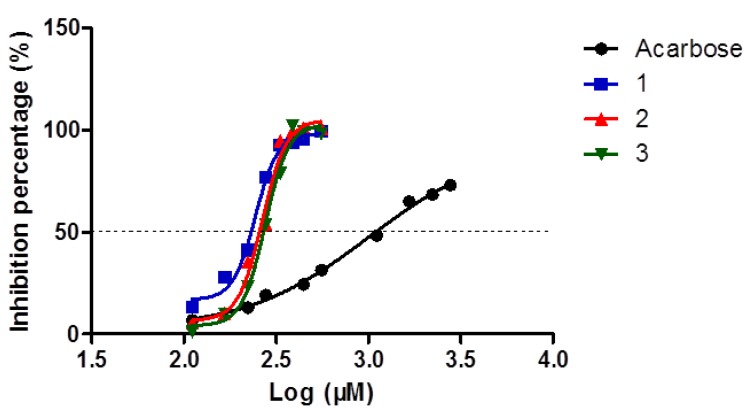
*α*-Glucosidase inhibitory effects of acarbose and three flavonoid glycosides: vitexin (**1**); isovitexin (**2**); isorhamnetin 3-*O*-*β*-d-rutinoside (**3**).

Since the inhibition is dependent on the concentration of substrate, enzyme and the duration of incubation with the enzyme, *α*-glucosidase inhibitory effects of the same compounds in different literature reports would be different. Previous studies showed considerable differences in IC_50_, even for the same compound (25.11, 9.48 and 420 μM for vitexin; 23.26, 15.49 and 1000 μM for isovitexin; 510 μM for isorhamnetin 3-*O*-*β*-d-rutinoside) compared to acarbose (IC_50_ = 228.16 μM) or other positive controls, respectively [10,19,20]. As a consequence, it was necessary to test the effects of these flavonoid glycosides, which are regarded as the major constituents of this herbal medicine, in parallel, even though their *α*-glucosidase inhibitory effects were reported before. The investigation into inhibitory effects of compounds **1**–**3** isolated from *Microctis Folium* was carried out using acarbose (IC_50_ = 1007 μM) as positive control. All of the results showed dose-dependent inhibition of *α*-glucosidase activity (Figure 3). The IC_50_ values for compounds **1**–**3** in this study were determined to be 244.0 μM (96.9 μg/mL), 266.2 μM (115.1 μg/mL) and 275.4 μM (172.0 μg/mL), respectively. Thus, vitexin, isovitexin and isorhamnetin 3-*O*-*β*-d-rutinoside might be potential *α*-glucosidase inhibitory candidates. 

The IC_50_ of the methanol extract (61.3 μg/mL) is higher than those of compounds **1**–**3** (96.9, 115.1 and 172.0 μg/mL, respectively). This might be caused by other materials in the extract, such as tannins, which can accelerate the sedimentation of proteins. Also, the possible interactions between compounds **1**–**3** or other components might show synergistic effects, which need further study. 

### 2.3. Optimization of HPLC Condition

**Figure 4:**
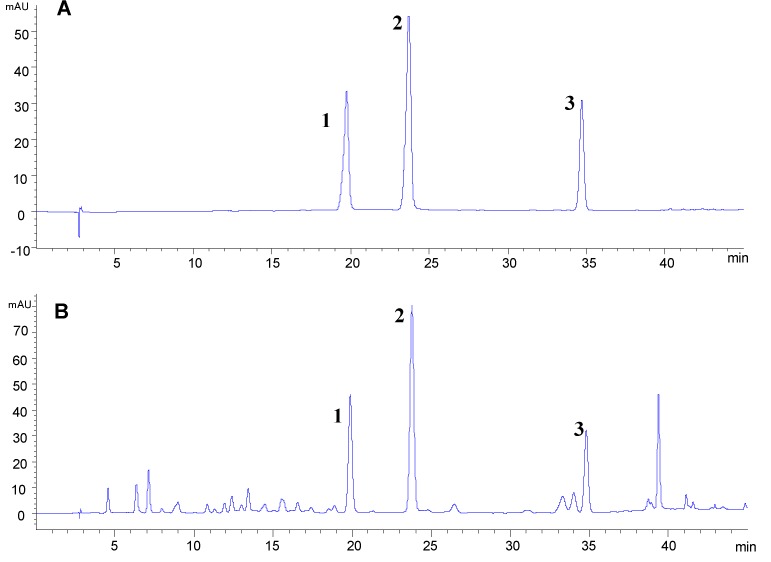
Typical HPLC-DAD chromatograms of the three flavonoid glycosides (**A**) and *Microctis Folium* (**B**). vitexin (**1**); isovitexin (**2**); isorhamnetin 3-*O*-*β*-d-rutinoside (**3**).

In order to obtain good resolution, different analytical columns, mobile phases and elution programs were tested using the mixed reference compounds and a leave sample BZY9, which was rich in compounds **1**–**3**. Finally, on a Grace Alltima C18 column (4.6 mm × 250 mm, 5 μm), the three flavonoid glycosides were eluted with baseline separation (Figure 4) under a gradient elution program of 0.1% phosphoric acid in water (A) and methanol (B). The gradient program is described in Section 3.7.

### 2.4. Optimization of Sample Extraction for HPLC Analysis

**Figure 5:**
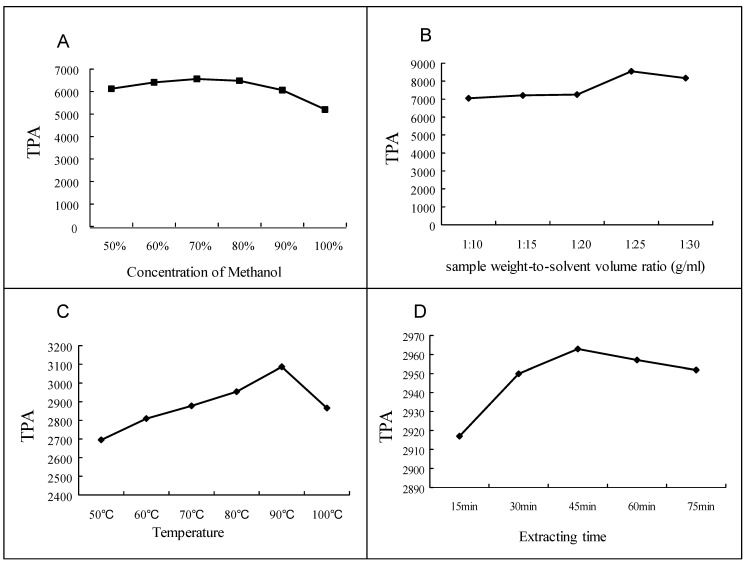
Results of optimization of sample extraction: assessment of methanol concentration (A); assessment of sample weight-to-solvent volume ratio (B); assessment of extraction temperature (C); assessment of extraction time (D).

A series of univariate extraction experiments were conducted on a Syncore Polyvap (Büchi, Flawil, Switzerland), Analyst and Reactor to assess the optimal extraction conditions, with the total peak areas (TPA) of the three flavonoid glycosides as the standard. The samples were extracted using methanol, ethanol and water, respectively, among which methanol showed best extraction efficiency. Then 60%, 70%, 80%, 90% and 100% aqueous methanol (*v*/*v*) were tested and the results revealed that 70% methanol was the most satisfactory solution (Figure 5A). Also, variations of sample weight-to-solvent volume ratio (*w*/*v*) were investigated, and a *w*/*v* of 1/25 g/mL was chosen since a greater volume of solvent did not show increased extraction efficiency (Figure 5B). Meanwhile extraction temperature was determined by comparing the efficiencies of extraction at 60, 70, 80, 90, 100 °C, respectively. At 90 °C, the highest extraction efficiency was achieved (Figure 5C). After comparison, 45 min was selected while identifying the influence of extracting time, rather than 15, 30, 45, 60 or 75 min (Figure 5D). The best extraction method was thus found to be refluxing at 90 °C for 45 min with 70% methanol in a ratio of 1/25 g/mL (*w*/*v*).

### 2.5. Method Validation

The developed HPLC method was validated for linearity, sensitivity, precision, stability, and accuracy. The data for retention times, calibration curves, test ranges, LOD and LOQ are presented in Table 1. The results indicated excellent linearities, with high correlation coefficients (R^2^ = 0.9999) within the test ranges. The relative standard deviations (RSDs) for intra- and inter-day repeatability were less than 0.50% and 0.74%, respectively. The corresponding stability study illustrated good stability of the method within 48 h. The accuracy, according to recovery, ranged from 96.0% to 103.6% (Table 2). The validation results suggested that the method developed in this paper was accurate and reliable for the quantitative analysis of the three flavonoid glycosides in *Microctis Folium*.

**Table 1:** Calibration curves and LODs, LOQs for the three flavonoid glycosides **1**–**3**.

Flavonoid glycosides	R_T_ (min)	Calibration curve ^a^	R^2^	Test range (µg/mL)	LOD ^b^ (ng)	LOQ ^c^ (ng)
Vitexin (**1**)	19.8	y = 27.707x + 29.277	0.9999	3.2–206.0	0.6	1.9
Isovitexin (**2**)	23.7	y = 21.677x + 20.783	0.9999	3.3–214.0	0.7	2.5
Isorhamnetin 3-*O*-β-d-rutinoside (**3**)	34.7	y = 12.942x + 14.820	0.9999	3.3–212.0	0.5	1.5

^a^ y = peak area and x = concentration (μg/ml); ^b^ Limit of detection (S/N = 3); ^c^ Limit of quantification (S/N = 10).

**Table 2:** Accuracy of HPLC method for the determination of the three flavonoid glycosides **1**–**3**.

	Original (mg)	Spiked (mg)	Found (mg)	Recovery ^a^ (%)	R.S.D (%)
Vitexin (**1**)		0.55	1.03	103.6	1.98
	0.46	0.50	0.94	96.0	1.19
		0.38	0.83	97.4	0.98
Isovitexin (**2**)		1.35	2.51	102.2	1.09
	1.13	1.10	2.24	103.6	0.99
		0.85	1.96	97.6	1.27
Isorhamnetin 3-*O*-*β*-d-rutinoside (**3**)		0.88	1.59	98.9	2.21
	0.72	0.74	1.44	97.3	1.00
		0.59	1.30	98.3	1.28

^a^ Recovery (%) = 100 × (amount found—original amount)/amount spiked. R.S.D (%) = 100 × S.D./mean.

### 2.6. Quantitative Analysis of Three Flavonoid Glycosides

Considering the significant antidiabetic activities and high contents of vitexin, isovitexin and isorhamnetin 3-*O*-*β*-d-rutinoside in *Microctis Folium*, it’s necessary to develop a reliable method to determine their contents. The contents of three flavonoid glycosides in different samples are shown in Table 3. The content of vitexin varied in the range of 0.10–2.31 mg/g based on dry weights. In this case, samples BZY1~3, BZY6 and BZY7 didn’t fulfill the requirement of Chinese Pharmacopoeia [12] which requires its content to be no less than 0.04%. The contents of isovitexin and isorhamnetin 3-*O*-*β*-d-rutinoside also varied amongst different samples, within the ranges of 1.17–5.65 mg/g and 1.66–5.16 mg/g, respectively. 

**Table 3:** The contents of the three flavonoid glycosides **1**–**3** (mg/g) in samples from different collections (n = 3) (Means ± SD).

	Vitexin	Isovitexin	Isorhamnetin 3-*O*-*β*-d-Rutinoside	Total contents of 3 flavonoid glycosides
BZY1	0.34 ± 0.00	1.80 ± 0.03	3.15 ± 0.03	5.30 ± 0.05
BZY2	0.20 ± 0.01	1.48 ± 0.03	3.18 ± 0.08	4.87 ± 0.11
BZY3	0.10 ± 0.00	1.17 ± 0.01	5.16 ± 0.01	6.42 ± 0.02
BZY4	0.61 ± 0.02	2.13 ± 0.05	2.55 ± 0.07	5.29 ± 0.13
BZY5	0.53 ± 0.00	1.97 ± 0.02	2.40 ± 0.01	4.90 ± 0.03
BZY6	0.30 ± 0.00	1.96 ± 0.02	4.01 ± 0.05	6.27 ± 0.07
BZY7	0.29 ± 0.01	1.43 ± 0.04	2.69 ± 0.06	4.41 ± 0.11
BZY8	1.32 ± 0.01	3.30 ± 0.02	3.06 ± 0.03	7.68 ± 0.06
BZY9	2.31 ± 0.04	5.65 ± 0.08	3.60 ± 0.05	11.55 ± 0.17
BZY10	0.46 ± 0.02	2.43 ± 0.04	2.39 ± 0.09	5.28 ± 0.14
BZY11	0.85 ± 0.04	1.73 ± 0.06	1.66 ± 0.06	4.23 ± 0.16

## 3. Experimental

### 3.1. Chemicals

*α*-Glucosidase from recombinant *Saccharomyces cerevisiae*, expressed in unspecified host and *p*-nitrophenyl *α*-d-glucopyranoside (PNPG) as the substrate of *α*-glucosidase and acarbose (≥95%) were purchased from Sigma (St. Louis, MO, USA). The solvents used for HPLC analysis were HPLC grade methanol and 85% phosphoric acid purchased from Merck (Darmstadt, Germany). The distilled water was purified with a Millipore Milli Q-Plus system (Millipore, Milford, MA, USA). All other chemicals were analytical grade.

### 3.2. Plant Materials

The *Microctis Folium* samples (BZY1-11) were collected from different places in Guangdong Province, China, and were authenticated by Professor Guang-Xiong Zhou from School of Pharmacy at Jinan University. All samples were dried in an oven at 60 °C for 24 h. The voucher specimens were deposited at the department of Institute of Chinese Medical Sciences, University of Macau, Macao SAR, China.

### 3.3. Extraction and Isolation

*Microctis Folium* (BZY9, 200 g) was extracted three times under reflux with 70% methanol (1.5 L) for 45 min. The extract was chromatographed on Sephadex LH-20 using methanol, followed by purification with Prep-HPLC to afford three flavonoid glycosides **1**–**3** applying isocratic elution (40%, 40%, 55% methanol, respectively) on a Grace Alltima C18 preparative column (10 μm, 250 mm × 22 mm, Grace-Alltech, Deerfield, IL, USA). Each compound was determined and identified by ^1^H-NMR, ^13^C-NMR and LC-MS analysis. The purities were determined to be all above 98% by normalization of the peak area detected using HPLC-DAD analysis.

### 3.4. Samples Preparation for α-Glucosidase Inhibitory Assay

The 70% methanol extract of BZY9, acarbose and three flavonoid glycosides of accurate quantities were dissolved with 50% DMSO and diluted to a series of suitable concentrations with 50% DMSO for *α*-glucosidase inhibition activity assay.

### 3.5. Assay of α-Glucosidase Inhibitory Activity

The inhibitory activity on *α*-glucosidase was performed on 96-well microplate [25]. A total of 100 μL reaction mixture containing 50 μL of 100 mM phosphate buffer (pH 6.8), 20 μL of 2.5 mM PNPG in the buffer, 20 μL of 2.4 U/mL *α*-glucosidase in phosphate buffer and 10 μL of investigated samples in the wells was thoroughly mixed. After incubation at 37 °C for 15min, 80 μL of 0.2 M sodium carbonate solution was added to each well to stop the reaction. Then the absorbance (A_S_) at 405 nm was recorded (Spectra Max M5 Microplate Reader, Molecular Devices LLC, Sunnyvale, CA, USA). The control sample was the mixture of solvent instead of the test sample. In sample blank and control blank, *α*-glucosidase was instead with buffer in the mixtures, respectively. The inhibition of test sample on *α*-glucosidase could be calculated as:

Inhibition= 1− (A_S_ − A_SB_)/(A_C_ − A_CB_)

where A_S_, A_SB_, A_C_, and A_CB_ are the absorbances of sample, sample blank, control, and control blank, respectively. The measurement was carried out in triplicate. 

### 3.6. Samples Preparation for HPLC Analysis

Each accurately weighed powder sample (0.40 g) was extracted with 10 mL of methanol-water (70: 30) mixture at 90 °C for 45 min, at 100 rpm on a Syncore Polyvap, Analyst and Reactor (Buchi Syncore, Flawil, Switerland). After cooling, the loss of solvent was made up to its original weight and then samples were blended together. Afterwards, the sample solution was filtered through a 0.45 μm Econofilter (Agilent Technologies, Santa Clara, CA, USA) and 10 μL of sample solution was injected into the HPLC system for analysis. Extraction and analysis of all the samples were carried out in triplicate and the results averaged.

### 3.7. Chromatographic Conditions

HPLC analysis was carried out using Agilent Series 1200 liquid chromatography (Agilent Technologies Inc.), containing a quaternary pump, a vacuum degasser, an auto-sampler, a column oven and photodiode array detector. The separation was completed on a Grace Alltima C18 analytical column (5 μm, 250 mm × 4.6 mm, Grace-Alltech). The column temperature was held at 35 °C with a flow rate of 1.0 mL/min. The binary gradient elution system consisted of 0.1% phosphoric acid in water (A) and methanol (B) was used, employing the following gradient: 0–10 min, 25%–35% B; 10–30 min, 35%–45% B; 30–45 min, 45%–80% B. The UV detection wavelength was set at 340 nm.

### 3.8. Method Validation of HPLC Analysis

The proposed HPLC method was tested to determine linearity, sensitivity, precision, stability, and accuracy. Accurately weighed reference compounds **1**–**3** were dissolved in an appropriate volume of methanol to produce corresponding stock standard solutions (206.0, 214.0, and 212.0 μg/mL for standards **1**–**3**, respectively). A series of standard working solutions were obtained by consecutive dilution of the stock solutions to get the calibration curves. Analysis of the standard working solutions were performed in triplicate and the results averaged. The calibration curves were plotted after linear regression of the peak areas *versus* concentrations. The sensitivity was conducted by the limit of detection (LOD) and limit of quantification (LOQ) which were determined under the present chromatographic conditions at a signal-to-noise (S/N) ratio of about 3 and 10, respectively. Intra- and inter-day variations were applied to evaluate the precision of this assay. They were completed by analyzing BZY9 (collected from Zhanjiang, Guangdong) in six replicates within one day and three consecutive days, respectively. The relative standard deviation (RSD) was used as the measurements of precision. The stability was tested by recording the peak areas of the three flavonoid glycosides at 0, 2, 4, 8, 12, 24 and 48 h. Spike recovery was applied to evaluate the accuracy of the assay as known amount of individual reference compounds were added into accurately weighed samples (BZY9). The mixtures were extracted and analyzed in triplicate using the method described in Section 3.6.

## 4. Conclusions

The 70% methanol extract of *Microctis Folium* and three flavonoid glycosides isolated from this plant material were investigated for their *α*-glucosidase inhibitory effects*.* The extract showed strong inhibitory activity against *α*-glucosidase, while vitexin, isovitexin and isorhamnetin 3-*O*-*β*-d-rutinoside all revealed satisfactory effects referred to positive control (IC_50_ = 1,007.0 μM). Thus, we propose that *Microctis Folium,* a herbal medicine containing a great amount of flavonoids, may be a useful food material for diabetic patients that warrants further investigation. For quality control of *Microctis Folium,* vitexin, isovitexin and isorhamnetin 3-*O*-*β*-d-rutinoside can be used as potential biomarkers and chemical markers due to their significant contents and bioactivities.

As it is vital to evaluate the quality of *Microctis Folium* for safety considerations and better commercial development of this herb, a simple and reliable HPLC-DAD method was developed for the quantification of these three flavonoid glycosides in *Microctis Folium* and applied successfully to determine the contents of these components in samples collected from different locations. 
